# IC-Tagging and Protein Relocation to ARV muNS Inclusions: A Method to Study Protein-Protein Interactions in the Cytoplasm or Nucleus of Living Cells

**DOI:** 10.1371/journal.pone.0013785

**Published:** 2010-11-02

**Authors:** Alberto Brandariz-Nuñez, Rebeca Menaya-Vargas, Javier Benavente, Jose Martinez-Costas

**Affiliations:** Department of Biochemistry and Molecular Biology, Faculty of Pharmacy and Center for Research in Biological Chemistry and Molecular Materials, University of Santiago de Compostela, Santiago de Compostela, Spain; University of Crete, Greece

## Abstract

**Background:**

Characterization of protein-protein interactions is essential for understanding cellular functions. Although there are many published methods to analyze protein-protein interactions, most of them present serious limitations. In a different study we have characterized a novel avian reovirus muNS-based protein tagging and inclusion targeting method, and demonstrated its validity to purify free an immobilized protein.

**Methodology/Principal Findings:**

Here we present a method to identify protein-protein interactions inside living eukaryotic cells (tested in primate and avian cells). When p53 was tagged with Intercoil (IC; muNS residues 477–542), it not only got integrated into muNS cytoplasmic inclusions, but also attracted its known ligand SV40 large T antigen (TAg) to these structures. We have also adapted this system to work within the cell nucleus, by creating muNS-related protein chimeras that form nuclear inclusions. We show that nuclear muNS-derived inclusions are as efficient as cytoplasmic ones in capturing IC-tagged proteins, and that the proteins targeted to nuclear inclusions are able to interact with their known ligands.

**Conclusions/Significance:**

Our protein redistribution method does not present the architectural requirement of re-constructing a transcription factor as any of the two-hybrid systems do. The method is simple and requires only cell transfection and a fluorescence microscope. Our tagging method can be used either in the cytoplasm or the nucleus of living cells to test protein-protein interactions or to perform functional studies by protein ligand sequestration.

## Introduction

Viroplasms, viral factories or virus inclusion bodies are different names given to the cellular compartments where most viruses carry out their morphogenesis. They are usually generated from one or several viral proteins that act as a scaffold or matrix, nucleating the inclusion that is formed by protein-protein interactions. The matrix proteins attract and concentrate the viral components, increasing the overall efficiency of the viral replication process [1], [2].

Avian reoviruses belong to the genus *Orthoreovirus*, family *Reoviridae* [3], [4] and constitute dangerous poultry pathogens [5], [6]. Details on their structure and replication cycle are available elsewhere [7], [8], [9]. Although these viruses replicate in the cytoplasm of infected cells, at least two viral proteins have been reported to display nuclear localization [10], [11]. In recent years, our laboratory has investigated the mechanisms that avian reoviruses use to produce viral factories. The results revealed that avian reovirus non-structural protein muNS is able to generate factory-like inclusions when expressed in different cell lines and using different expression systems, suggesting that this protein forms the matrix of the factories in infected cells [12]. Additionally, muNS attracts other viral proteins in a specifically and temporally controlled way, thus contributing to regulate the morphogenesis of the viral particle [13]. In a recent study we have demonstrated that avian reovirus inclusion formation does not depend on the cytoskeleton, and that avian reovirus factories and muNS-derived inclusions are not microtubule-associated [14]. An analysis of the inclusion-forming capability of muNS truncations revealed that the minimal muNS portion able to generate intracellular inclusions comprises its C-terminal one third (residues 448–635). We designated it muNS-Mi, and characterized the role that its four different domains (Coil1, Coil2, Intercoil and C-Tail) play in inclusion formation. Most notably, we were able to demonstrate that Coil1 region (residues 448 to 477) can be replaced by a dimerization domain, and that the C-Tail domain (residues 605–635) orients muNS inter-monomer contacts to generate basal oligomers that dictate the inclusion shape and inclusion-forming efficiency [14]. In the same study, we developed a simple protocol for the purification of the inclusions made by muNS in baculovirus-infected cells.

Based on the results obtained, and in a different study (manuscript in preparation), we developed a method to target foreign proteins to the muNS-related inclusions in recombinant baculovirus-infected insect cells. It is based on the strong affinity between muNS-derived inclusions and the 66 residue-long, Intercoil domain (IC, muNS residues 477–542). Thus, tagging proteins with IC caused their re-localization to the muNS-derived inclusions. Using a method that we had previously designed for the purification of muNS-derived inclusions [14], we developed a protocol for purification of foreign proteins that had been tagged with the IC domain. We demonstrated that the inclusion-targeted proteins were active either when integrated in the inclusions, or after their solubilization and separation from the muNS-related inclusions. Our study also showed that the inclusion-integrated proteins were active both in vitro and in vivo [25].

In the present study we demonstrate that our IC-tagging and inclusion-targeting method works as well in transfected cells of avian and mammalian origin. We also show that the cellular protein p53, in spite of being a nuclear protein, is relocated to cytoplasmic muNS-related inclusions by IC-tagging. Additionally, when p53 gets relocated to the inclusions, it attracts the SV40 Large T antigen (TAg). We showed our protocol to work in two different cell lines of different origin: COS-7, a mammalian cell line expressing TAg endogenously, and CEF (avian) in which we expressed TAg by plasmid transfection. The method described in this study can indifferently use any of the four different muNS-related proteins as the inclusion-forming unit: muNS, muNS-Mi, GFP-muNS and GFP-muNS-Mi, and all of them efficiently capture the IC-tagged proteins. Furthermore, we have also adapted this highly versatile system to work within the cell nucleus, by creating muNS protein chimeras that are able to form nuclear inclusions. We showed that nuclear inclusions are as efficient as cytoplasmic ones in capturing IC-tagged proteins, and that the proteins targeted to nuclear inclusions are perfectly able to interact with their known ligands. The nuclear inclusion environment represents an ideal means for studying the interactions between proteins that normally reside at the cell nucleus. Also, it opens the possibility of using them for capturing or sequestering nuclear proteins, and thus removing them from their normal intra-nuclear localization for functional studies.

## Results

### IC-directed targeting to muNS-related inclusions for detecting intracellular protein-protein associations in living cells

**Figure 1:**
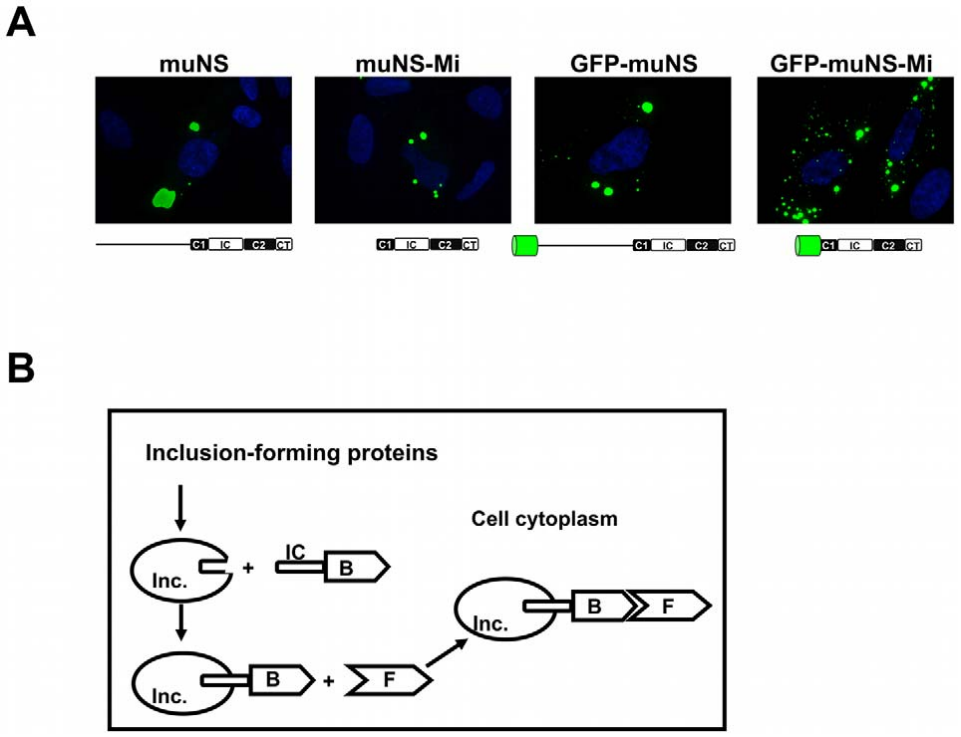
Strategy for detecting protein-protein interactions based on muNS-derived inclusion targeting by IC tagging. **A.** CEF cells were transfected with plasmids expressing the proteins indicated on top of the pictures. The cells were immunostained for muNS (green), except those expressing GFP that were visualized without antibodies. Nuclei were counterstained blue with DAPI. A schematic representation of the muNS-derived constructs is depicted below each picture, where the previously described muNS-Mi domains (Coil 1 or C1; Intercoil or IC; Coil2 or C2, and C-Tail or CT, [14]) are shown in boxes. The green barrel represents GFP. **B.** Principle of the method. Plasmids expressing a muNS-derived inclusion-forming protein and an IC-tagged bait protein (B) are cotransfected into cells. The IC-B protein should be incorporated into inclusions and should recruit a bait-interacting fish protein (F) into these structures. The F protein could be an endogenous protein or a plasmid-expressed protein.

Previously [25] we have demonstrated that the avian reovirus muNS IC domain is a suitable tag for targeting fused proteins to the inclusions formed by different muNS versions: muNS-Mi, GFP-muNS and GFP-muNS-Mi (Figure 1A and manuscript in preparation). In the present work we investigated whether IC-tagging could also be employed for detecting protein-protein interactions within living cells, by using the strategy outlined in Figure 1B. In brief, a plasmid expression vector carrying the gene encoding the muNS-derived protein is co-transfected into cells with a plasmid expressing a IC-tagged protein B (bait) that should be incorporated into the inclusions. A third plasmid expressing a protein F (fish) that is thought to interact with the bait is also transfected and, if B and F interact, F would be also attracted to the inclusions by its interaction with B. In contrast, if B and F do not associate, F will retain its normal intracellular distribution in the transfected cell. The localization of both proteins can be easily visualized by immunostaining with specific antibodies.

### Ability of the different inclusion-competent muNS versions to recruit p53-IC

**Figure 2:**
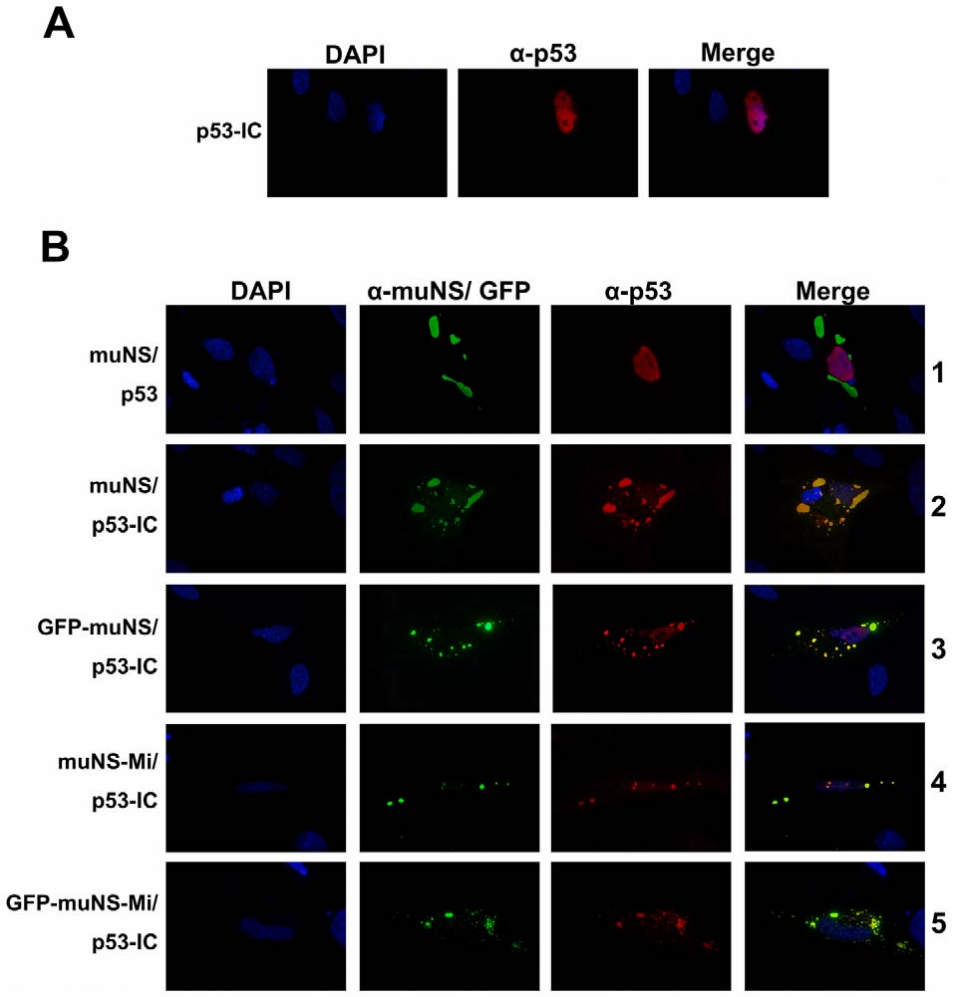
Intracellular distribution of p53 and p53-IC in the presence of muNS-derived cytoplasmic inclusions. **A.** Intracellular distribution of p53-IC in single-transfected CEF cells. p53 is immunostained red and nuclei are counterstained blue with DAPI. **B.** CEF cells were cotransfected with the plasmids expressing the proteins indicated on the left of the figure. Cells were immunostained with rabbit anti-muNS antibodies (muNS, green) and mouse anti-p53 monoclonal antibody (p53, red). The constructs containing GFP were visualized without antibodies (green). Nuclei were counterstained blue with DAPI.

To test the general utility of this technology, we took advantage of the well-defined interaction between the mammalian tumor suppressor protein p53 and the SV40 tumor protein TAg [15]. Thus, we decided to tag p53 with IC and check its ability to re-localize and recruit the SV40 TAg to muNS-related cytoplasmic inclusions. We used a plasmid that expresses the IC-tagged p53 protein [25] (manuscript in preparation) and first analyzed its localization in transfected CEF cells by immunofluorescence. Both the untagged (not shown) and IC-tagged p53 (Figure 2A) localized primarily to the nucleus, showing that IC-tagging did not affect the normal localization of p53. Next, we tested the ability of the IC tag to target p53 to muNS-derived inclusions by co-transfection. In the presence of muNS, untagged p53 localized primarily in the nucleus with no visible co-localization with muNS inclusions (Figure 2B, row 1). This result demonstrates that p53 does not associate with muNS inclusions on its own, and also that the p53-specific antibody used for immunostaining does not cross-react with muNS inclusions. In contrast, IC-tagged p53 localized almost exclusively to muNS inclusions in co-transfected cells (Figure 2B, row 2). These results demonstrate that incorporation of p53-IC into muNS inclusions did not disrupt inclusion formation and that our inclusion-targeting system works also with nuclear proteins. Exactly the same results were obtained when using all the other inclusion-forming proteins described in Figure 1 in the co-transfection experiments. Thus, the inclusions formed by GFP-muNS (Figure 2B, row 3), muNS-Mi (Figure 2B, row 4) or GFP-muNS-Mi (Figure 2B, row 5), all attracted the IC-tagged p53 that was completely re-localized to the respective inclusions. In contrast, untagged p53 showed no co-localization with any of the inclusions and remained in the nucleus in the co-transfection experiments (Figure 2B, row 1 and data not shown).

Interestingly, in most cells co-expressing muNS-Mi and p53-IC we observed the presence of both cytoplasmic and nuclear inclusions (Figure 2B, row 4). However, only cytoplasmic inclusions were detected in cells co-expressing GFP-muNS-Mi and p53-IC (Figure 2B, row 5). We hypothesized that, due to its small size, a fraction of the muNS-Mi protein is translocated to the nucleus in association with p53-IC, and that nuclear muNS-Mi is still able to form inclusions and to recruit IC-tagged p53.

### Association of p53 with endogenous SV40 T-Antigen (TAg) in the cytoplasm of COS-7 cells

**Figure 3:**
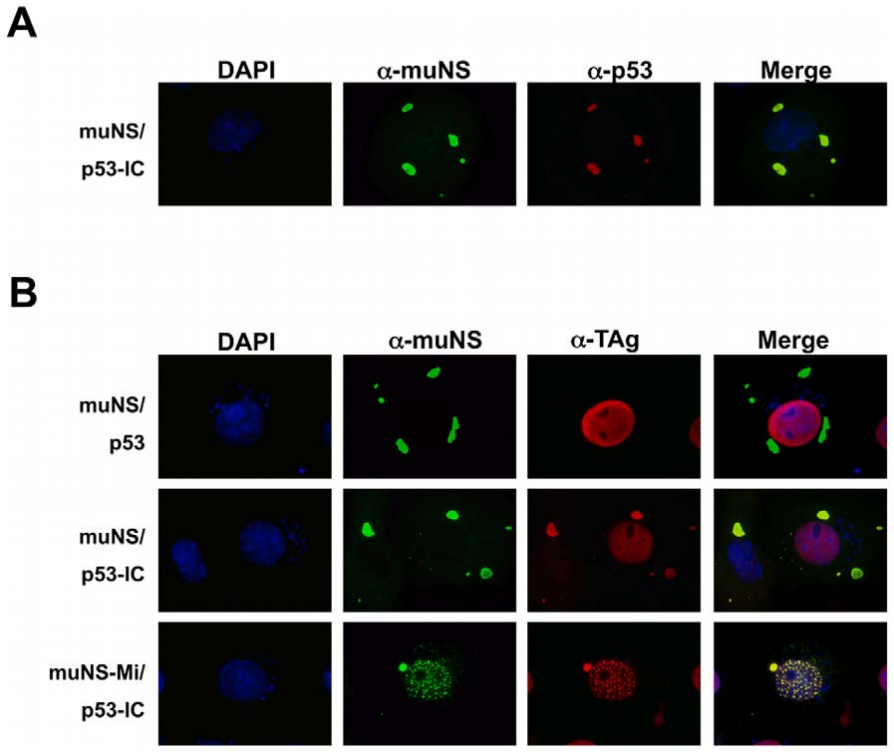
Intracellular distribution of endogenous TAg in COS-7 cells expressing inclusion-competent proteins and p53/p53-IC. **A.** Intracellular localization of muNS (green) and p53-IC (red) in cotransfected COS-7 cells. Nuclei were counterstained blue with DAPI. **B.** COS-7 cells were co-transfected with plasmids expressing the proteins indicated at the left of the figure. The cells were immunostained with rabbit anti-muNS antibodies (muNS, green) and mouse anti-TAg (TAg, red). Nuclei were counterstained blue with DAPI.

We next decided to study whether p53-IC is able to recruit TAg to muNS-derived inclusions. First, we used COS-7 cells, because these cells constitutively express endogenous TAg, which largely localizes to the nucleus [16], [17]. As expected, and in agreement with the results obtained in CEF, in co-transfected COS-7 cells expressing p53-IC and any of the muNS-related inclusion-competent units (muNS, GFP-muNS, muNS-Mi and GFP-muNS-Mi), tagged p53 was localized almost exclusively in association with inclusions (Figure 3A and data not shown). In cells expressing muNS-Mi and p53-IC we again observed the formation of nuclear inclusions, as in CEF cells (Figure 2B, row 4 and data not shown). In contrast, untagged p53 localized to the nucleus, showing no co-localization with inclusions (not shown). These results confirm that our inclusion-targeting system works well in different cells types (CEF, COS-7 and Sf9; [25].

We next analyzed the intracellular distribution of TAg in COS-7 cells co-expressing p53 and an inclusion-forming protein. TAg localized to the nucleus, and did not co-localize at all with inclusions, when co-expressed with untagged p53 (Figure 3B, upper row and data not shown). These results indicated that TAg does not independently associate with inclusions and that the TAg-specific antibody used for immunostaining does not cross-react with the muNS and GFP moieties of the inclusion-forming protein. However, when we used IC-tagged p53, a significant portion of TAg was found in association with cytoplasmic inclusions (see Figure 3B, middle row). The same results were obtained with all four described inclusion-forming proteins (Figure 3B, bottom row and data not shown). We again observed some nuclear inclusions, in addition to cytoplasmic ones, when using muNS-Mi as inclusion-forming unit (Figure 3B, bottom row). The nuclear inclusions observed in COS-7 cells were more numerous than the ones previously observed in CEF, probably because the endogenous TAg of COS-7 cells helps p53 in towing muNS-Mi to the nucleus.

### Association of p53 with transiently expressed TAg in the cytoplasm of CEF cells

**Figure 4:**
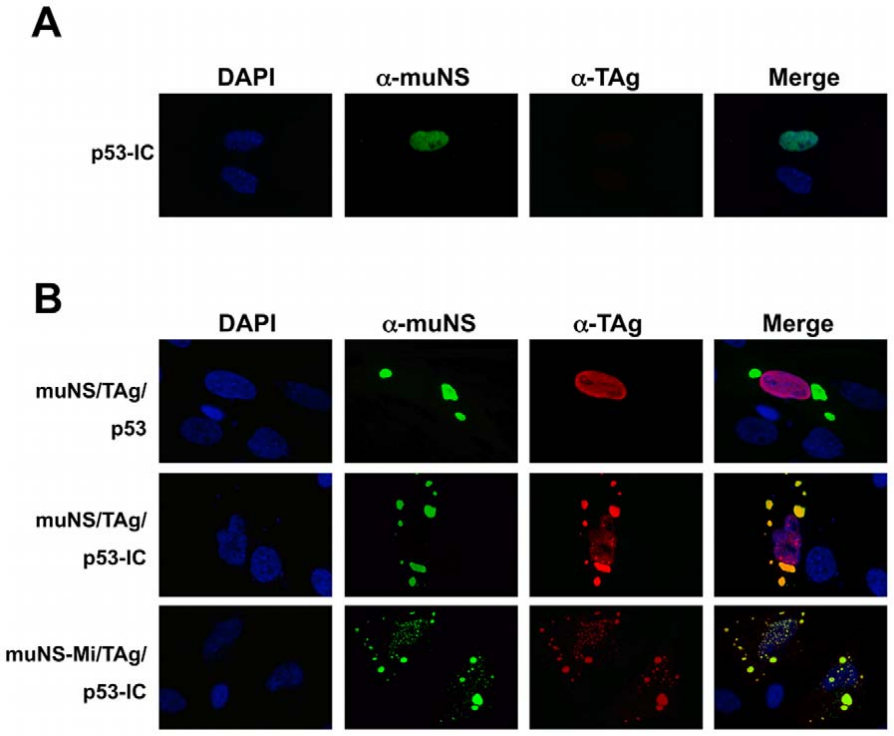
Intracellular distribution of plasmid-expressed TAg in CEF cells co-expresing inclusion-competent proteins and p53/p53-IC. **A.** CEF cells were transfected with the plasmid expressing p53-IC and they were subsequently immunostained with antibodies against muNS (green) and TAg. Nuclei were counterstained blue with DAPI. **B.** CEF cells were co-transfected with plasmids expressing the proteins indicated at the left of the figure. The cells were immunostained with antibodies against muNS (green) and anti-TAg (red). Nuclei were counterstained blue with DAPI.

So far, we have demonstrated the utility of our system for detecting the interaction between plasmid-expressed p53 and endogenous TAg in double-transfected COS-7 cells. Next, we wanted to demonstrate the validity of our method for detecting the interaction between two plasmid-expressed proteins in triple-transfected cells. First, to check that the TAg specific antibody does not cross-react with p53, we expressed p53-IC and examined by immunofluorescence whether it was recognized by antibodies against muNS and TAg. As expected, p53-IC could not be detected using the TAg-specific antibody, but was visualized in the nucleus of the transfected cells by reaction with muNS specific antiserum (Figure 4A). Next, we focused in localizing the intracellular distribution of TAg in triple-transfected CEF. TAg was localized exclusively in the nucleus of CEF cells co-expressing untagged p53 and an inclusion-forming protein (Figure 4B, upper row, and data not shown). However, when co-expressed with p53-IC, TAg localized mostly in cytoplasmic muNS inclusions, showing that our inclusion-targeting system works perfectly in different cell types and with different expression systems (Figure 4B, middle row). When muNS-Mi was used as inclusion-forming protein, again some nuclear inclusions were evident in the transfected cells, and they successfully attracted both tagged p53 and TAg (Figure 4B, bottom row).

### Engineering muNS-related proteins to form protein inclusions in the nucleus

**Figure 5:**
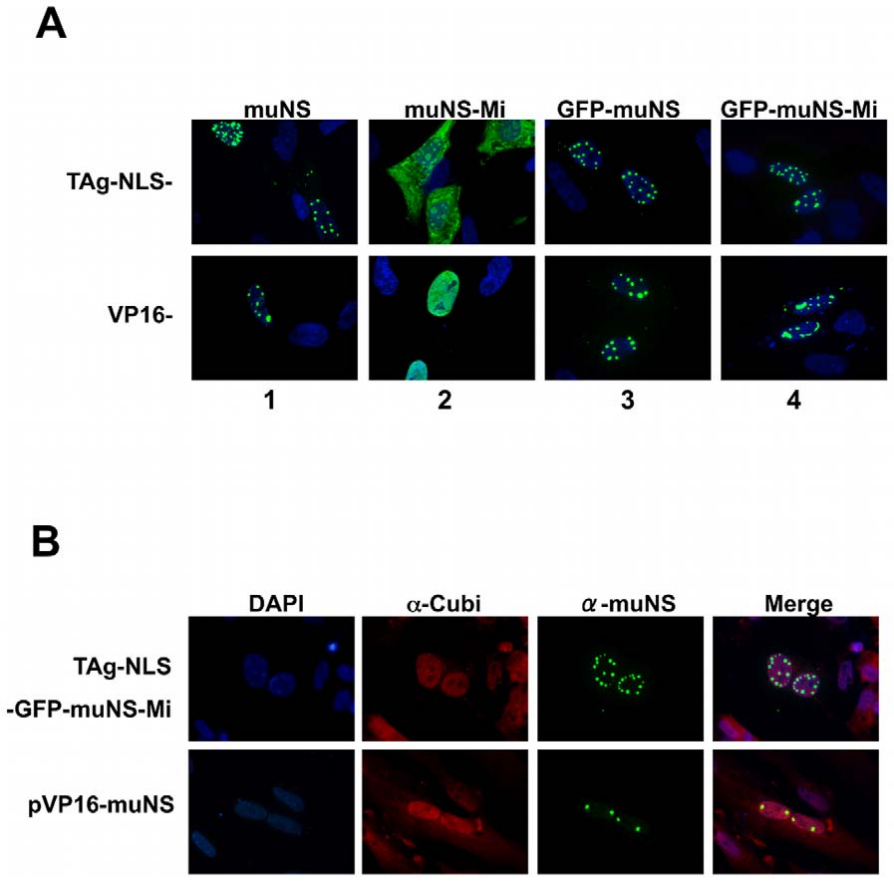
Subcellular localization of muNS-derived proteins containing nuclear localization sequences. **A.** CEF cells were transfected with plasmids expressing the chimeras formed by fusing the proteins indicated on top of the figure with the tags indicated on the left. The cells were immunostained for muNS (green), except those containing GFP that were visualized without antibodies (green). Nuclei were counterstained blue with DAPI. **B.** CEF cells were transfected with plasmids expressing the proteins indicated at the left of the figure and immunostained with antibodies against muNS (green) and conjugated ubiquitin (red). The construct containing GFP was visualized without antibodies (green). Nuclei were counterstained blue with DAPI.

Our finding that nuclear inclusions are formed in cells co-expressing p53-IC and muNS-Mi prompted us to investigate the possibility of adapting our system for detecting protein-protein interactions within the cell nucleus, which would make our system more suitable for detecting associations between nuclear proteins. For this, we tried to generate nuclear-inclusion-forming proteins by fusing nuclear localization sequences (NLS) to muNS-related proteins in order to see if they could reach the nucleus and generate inclusions there. We chose the TAg nuclear localization sequence PKKKKKV [17] as a short, previously characterized NLS to be added to the N terminus of the different inclusion-forming proteins. Additionally, we also considered using a bigger domain for providing a functional NLS instead of the short sequences that could be not properly folded in the chimeric proteins due to their small size. Thus, we used the VP16 Herpes virus domain with a fused TAg NLS, that is part of the Mammalian Matchmaker Two-Hybrid Assay Kit (Clontech, Saint Germain en Laye, France), and fused it to the N-terminus of the four different muNS variants. Fluorescence microscopy analysis of the intracellular distribution of the fused constructs revealed that muNS, GFP-muNS and GFP-muNS-Mi produced nuclear inclusions when fused to either TAg NLS or VP16 (Figure 5A, columns 1, 3 and 4). Strikingly, the chimeras containing fused muNS-Mi did not generate inclusions (Figure 5A, column 2), but were distributed diffusely either throughout the cytoplasm (when fused to TAg NLS), or throughout the nucleus (when fused to VP16).

To rule out the possibility that any of the nuclear-inclusion-forming chimeras were substantially misfolded and/or targeted for degradation by the ubiquitin-proteasome system, the cells expressing the nuclear inclusion-competent constructs were immunostained with MAB FK2, which recognizes conjugated ubiquitin [18]. None of the constructs co-localized with conjugated ubiquitin, suggesting that they were forming inclusions by specific interactions between monomers and not by simple aggregation (Figure 5B, and data not shown).

### IC-tagged proteins are targeted to nuclear inclusions

**Figure 6:**
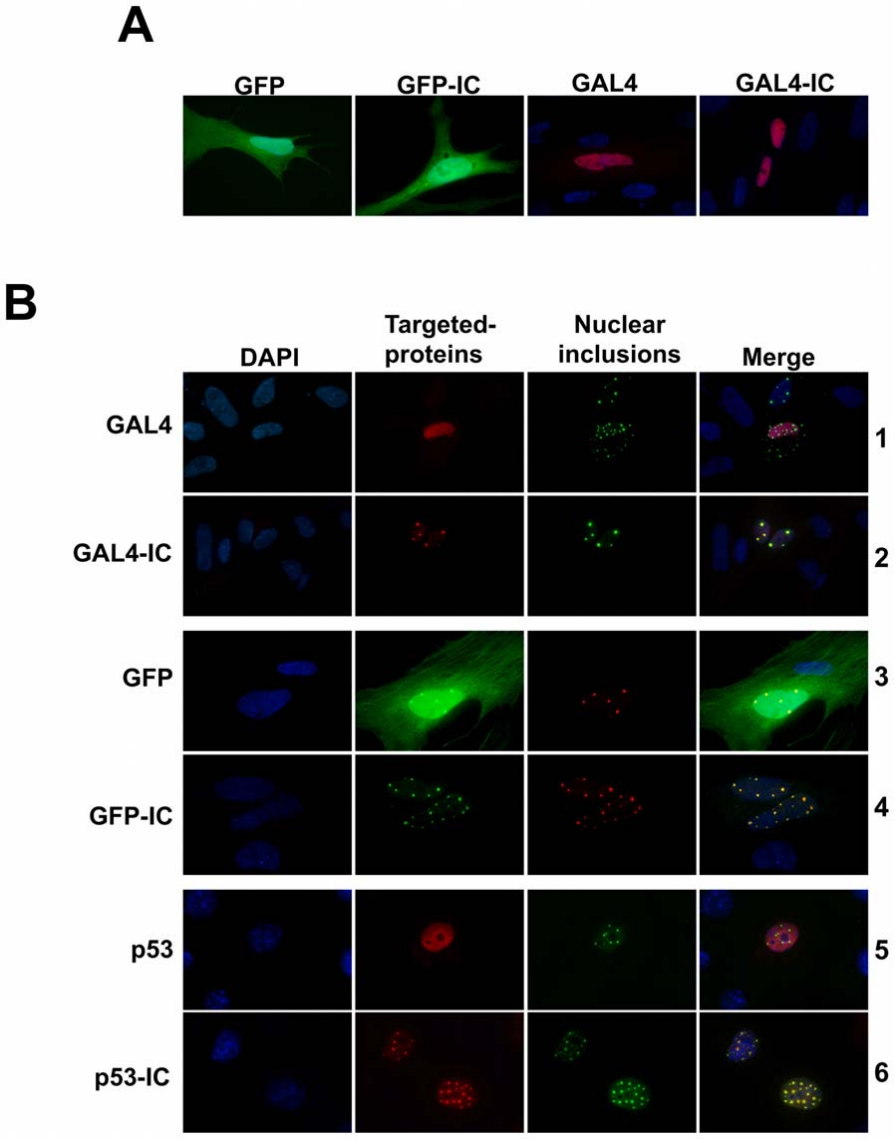
Intracellular distribution of GFP/GFP-IC, GAL4/GAL4-IC and p53/p53-IC in cells expressing nuclear inclusion-competent proteins. **A.** CEF cells were transfected with the plasmids expressing the proteins indicated on top of the figure and immunostained for GAL4 (red) except those containing GFP that were visualized without antibodies (green). Nuclei were stained with DAPI (blue). **B.** CEF cells were co-transfected with plasmids expressing the proteins indicated on the left of the figure and nuclear inclusion-competent, muNS-derived proteins. The targeted proteins were visualized in red with the exception of GFP (green) and the inclusions in green, with the exception of those in cells co-expressing GFP, where they were stained in red. Nuclei were counterstained with DAPI (blue).

To demonstrate that muNS-derived nuclear inclusions are able to recruit IC-tagged proteins, we constructed plasmids that express IC fused both to the C terminus of the nuclear protein GAL4 DNA binding domain, and to a protein that distributes uniformly throughout the whole cell (GFP). Fluorescence microscope analysis of single-transfected CEF cells revealed that IC-tagging does not change the normal intracellular distribution of any of the two proteins; GAL4-IC was evenly distributed throughout the nucleus, whereas GFP-IC remained distributed throughout the whole cell (Figure 6A). The distribution of the two untagged proteins did not change upon co-expression with muNS-derived nuclear-inclusion-forming proteins (Figure 6B, rows 1 and 3), indicating that neither of these proteins interact with inclusions and that the antibody against GAL4 does not cross-react with the inclusion-forming proteins. The same was true for p53-IC that was used on the experiments shown in Figures 3 and 4 (Figure 6B, row 5). In contrast, the three proteins were found to collect into muNS-derived nuclear inclusions when tagged with IC (Figure 6B, rows 2, 4 and 6), showing that our IC-tagging and inclusion association method is also able to target nuclear and nonnuclear proteins to nuclear inclusions. The same results were reproduced with all the nuclear-inclusion-forming proteins shown in Figure 5 (not shown), including those that produce fluorescent inclusions. These results further showed that the incorporation of IC-tagged proteins into muNS-derived nuclear inclusions does not disrupt inclusion formation. Finally, inclusion-associated GFP is properly folded since it continues to emit fluorescence.

### Association of p53 with SV40 TAg within the nucleus

**Figure 7:**
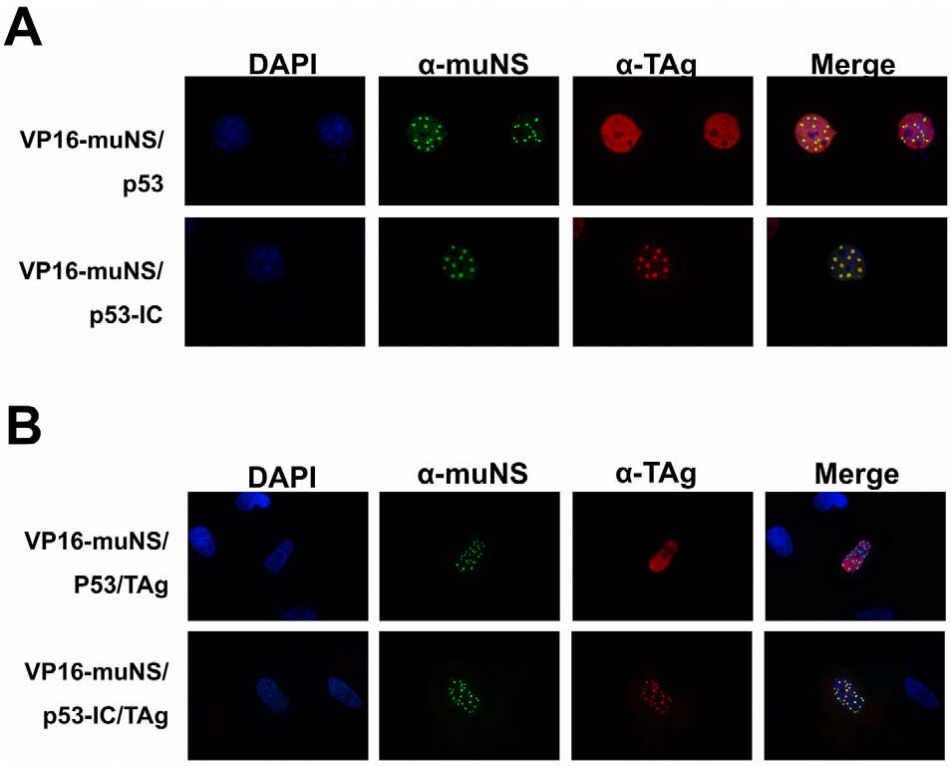
Intracellular distribution of TAg in cells expresing VP16-muNS and p53 or p53-IC. Cells were transfected with the plasmids expressing the proteins indicated on the left of the figure and immunostained with anti-muNS antibodies (green) and anti-TAg (red). Nuclei were stained with DAPI (blue). **A.** COS-7 cells. **B.** CEF cells.

Once demonstrated that nuclear muNS-derived inclusions are able to recruit IC-tagged proteins, we assessed the validity of our system to detect protein-protein interactions within the nucleus. For this, we examined again the interaction of p53 with SV40 TAg in either COS-7 cells (endogenous TAg) or CEF cells (plasmid-driven expression of TAg). First, we verified that p53-IC is targeted to nuclear inclusions in COS-7 cells as we have just shown for CEF (not shown). We next investigated the intracellular distribution of endogenous TAg in each of the two co-transfected cells. TAg was diffusely distributed throughout the nucleus of COS-7 cells co-expressing p53 and VP16-muNS (Figure 7A, upper row), indicating that TAg does not associate with nuclear inclusions and that the TAg-specific antibody does not cross-react with muNS-derived inclusions. In contrast, TAg re-localized to nuclear inclusions in cells expressing p53-IC (Figure 7A, bottom row), demonstrating that our method is able to detect the interaction of plasmid-expressed p53 with endogenous TAg in the nucleus of COS-7 cells.

The same results were obtained in triple-transfected CEF cells, when TAg was provided by plasmid expression. As in COS-7 cells, TAg does not associate with muNS-derived nuclear inclusions in CEF (Figure 7B, upper row). Only in cells expressing p53-IC, but not in those expressing untagged p53, plasmid-expressed TAg localized to nuclear inclusions (Figure 7B, bottom row), indicating that our method is also able to detect the interaction within the nucleus of two transiently expressed proteins.

The interaction p53-TAg in the two cell types was successfully detected when using each of the different nuclear-inclusion-competent muNS chimeras shown in Figure 5 (data not shown), indicating that several different inclusion-forming proteins, including fluorescent proteins, can be used in our system.

### Simultaneous detection of p53 and TAg in muNS-derived inclusions

**Figure 8:**
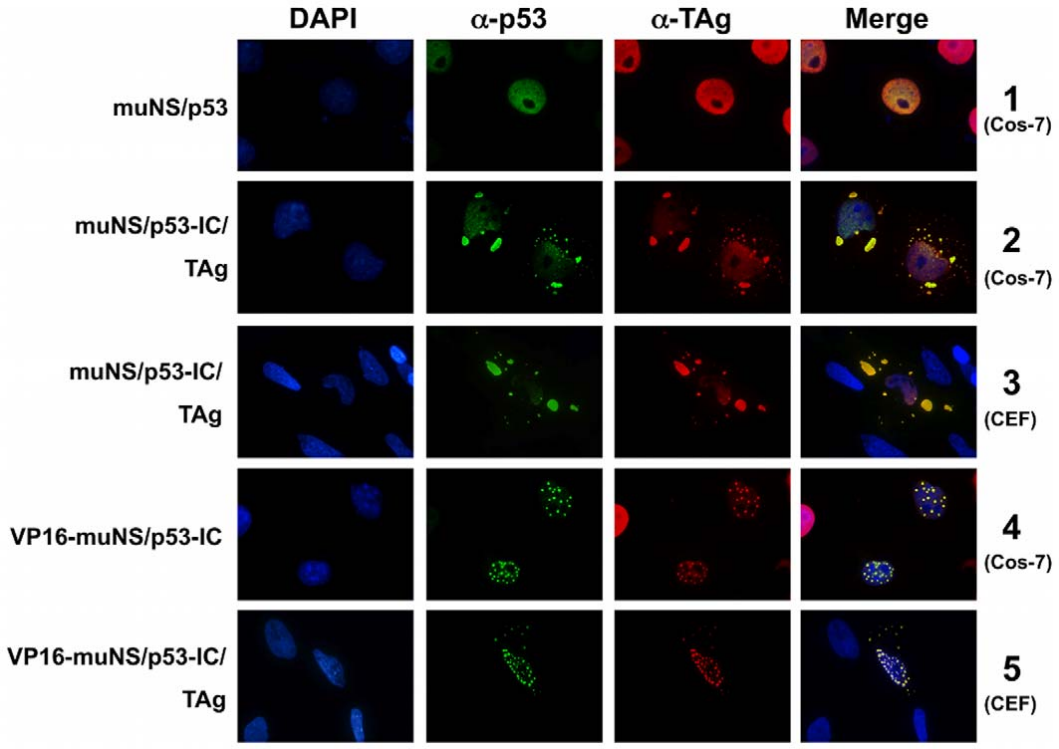
Intracellular distribution of TAg and p53-IC in the presence of cytoplasmic and nuclear inclusions. Cells were transfected with the plasmids expressing the proteins indicated on the left of the figure and immunostained with anti-p53 (green) and anti-TAg (red) antibodies. Nuclei were stained with DAPI (blue). Cell types are indicated in brackets on the right of the figure.

To conclusively demonstrate the presence of both interacting proteins inside muNS-derived inclusions, we performed double labeling experiments using specific antibodies against p53 and TAg. Once verified that there is no cross-reaction between both antibodies (Figure 4A and Figure 8, lane 1), we were able to simultaneously detect IC-tagged p53 and TAg inside cytoplasmic and nuclear muNS-derived inclusions in COS-7 (Figure 8, lanes 2 and 4) and CEF cells (Figure 8, lanes 3 and 5). Similar results were obtained with all other inclusion-forming constructs described in this study (not shown).

## Discussion

In a previous study we have characterized a simple and inexpensive method for purifying proteins expressed in baculovirus-infected cells. The method is based on: i) the ability of different avian reovirus muNS versions to form cytoplasmic inclusions that can be easily purified; and ii) the ability of these inclusions to recruit proteins tagged with muNS domains, with IC being the most effective domain [25]. In this study we have extended the applicability of this method for detecting protein-protein interactions within eukaryotic cells of avian and mammalian origin.

Our method is very simple and inexpensive, since only requires transient expression of the test proteins and a regular fluorescence microscope. It is also highly versatile, because it works successfully in different cell lines (avian and mammalian), can be used to detect protein-protein interactions in the nucleus or the cytoplasm, and can use several different inclusion-forming proteins, some of which are auto-fluorescent thereby facilitating inclusion detection. The versatility of our method also permits detection of interactions between nuclear and non-nuclear proteins in either the nucleus or the cytoplasm, as well as the interaction between two plasmid-expressed proteins or between a plasmid-expressed protein and an endogenous protein. Its main limitation resides on the availability of specific antibodies, but this can also be circumvented with the use of tagged problem proteins.

Our finding that an endogenous nuclear protein like COS7 TAg can be relocated to p53-IC-containing cytoplasmic inclusions suggests that our method might also be used for removing a protein from its normal nuclear localization, which would allow studying the effect that its loss of nuclear function has on cellular processes. On the other hand, to facilitate the detection of the interaction between nuclear proteins, we have adapted our method for detecting interactions inside the nucleus of eukaryotic cells by adding nuclear targeting sequences to the muNS-derived inclusion-forming proteins. Nuclear inclusions were efficient for both capturing IC-tagged proteins and detecting the interaction between p53-IC and TAg within the nucleus, their natural environment.

There are several published methods aimed at studying protein-protein interactions inside living cells of higher eukaryotes [19], [20], being the mammalian two-hybrid system the most widely used. However, we believe that the two-hybrid system has several disadvantages in comparison with our method. The first limitation of the two-hybrid system is that it can only be used to detect interactions between two plasmid-expressed proteins, but not the association of a plasmid-expressed protein with an endogenous protein. The second limitation is that the two-hybrid system can only detect interactions between two tagged proteins, whereas our method only requires the expression of one tagged protein. Furthermore, the two-hybrid system can only detect protein-protein interactions within the nucleus, and this may not work out well with cytoplasmic proteins. Finally, in the two-hybrid system the association of a protein A-containing activating domain with a protein B-containing DNA-binding domain has to reconstitute a functional transcription factor for successfully detecting the A-B interaction. This strategy has an obvious architectural disadvantage, since a positive result depends on the geometry of the association between two tagged proteins, because A-B association could occlude/inactivate the activating and/or the DNA-binding domains. However, in our method the geometry of the A-B association is irrelevant for its detection.

Recently, a mammalian reovirus muNS-derived platform for visualizing protein associations inside cells was developed and termed PIP (protein interaction platform; [21]). Using the mammalian reovirus muNS instead of the avian was not the only difference with our method, rather the whole concept is different. PIP does not target tagged proteins to an inclusion formed by a different polypeptide, but fuse together the bait protein and the minimal inclusion-forming portion of muNS, and wait for the fish polypeptide to be recruited to the bait-containing inclusion. This platform was subsequently adapted for the visualization of interacting proteins in yeast [22]. The authors claim that PIP is better and more efficient in yeast than the two-hybrid system, because it is able to detect more chaperone-effector interactions [22]. This is probably due to the lack of geometric restrictions of PIP relative to the two-hybrid system.

However, the mammalian reovirus muNS-derived PIP method presents some disadvantages that the authors acknowledge, and that are solved to a big extent with our inclusion-targeting protocol. For instance, PIP relies on the inclusion-forming ability of a bait-muNS fusion, and that fusion could alter the folding and/or activity of the fused partners. The fused bait may ablate the inclusion-forming ability of the reoviral protein, so that the bait-muNS fusion does no longer form inclusions. In support of this, we have found that minor alterations in the sequence of some inclusion-competent muNS versions leads to either abrogation of inclusions or the generation of amorphous aggregates instead of regular inclusions (Figure 5; [14]). On the other hand, the fused multimeric reoviral protein, which intertwines to create a big protein inclusion, might alter the proper folding of the bait partner. Our inclusion targeting protocol is less likely to have this problem. First of all, the small size of the IC tag to which the bait protein is fused should not significantly modify the native conformation of the bait. Second, the bait protein does not interact directly with inclusions, but through the IC tag, and we have shown that such association does not disrupt the capacity of our muNS-derived proteins to form inclusions. Furthermore, our method should be versatile enough to solve folding problems, since it can use different muNS-derived inclusion-forming units. An additional advantage of our method is that it has been adapted to visualize protein associations inside the nucleus, which should be very useful for detecting interactions between nuclear proteins in their normal environment, increasing the probabilities of success. The generation of nuclear compartments where particular proteins could be recruited for functional studies is other possible advantage of our system.

We are aware that our method presents the limitations of any other tagging method, since tag fusion could interfere with the proper folding of the bait protein. However, the extremely positive and reliable results obtained with the IC tag in the recruiting experiments described here and in a previous study [25], suggest that IC probably has a very compact and independent folding. Additionally, its small size makes it less likely to interfere with the bait protein folding. In the present and an additional study [25] we show examples where the IC-tagging and inclusion-targeting system successfully works with many different proteins, with different intracellular localizations, expressed in cells of different origins and with different expression systems. In all shown examples, the tagged protein remained active and/or associated with its known interacting partner.

## Materials and Methods

### Cells and antibodies

Primary cultures of chicken embryo fibroblasts (CEF) were prepared from 9- to 10-day-old chicken embryos [23] and grown in monolayers in medium 199 (Invitrogen, Barcelona, Spain) supplemented with 10% (w/v) tryptose-phosphate broth and 5% (v/v) calf serum. COS-7 cells [16] were grown in monolayers in medium D-MEM supplemented with 10% fetal bovine serum. Rabbit polyclonal antiserum against avian reovirus S1133 muNS protein was raised in our laboratory [12]. Monoclonal antibody PAB40 specific for p53 was obtained from Sigma-Aldrich (Madrid, Spain). Monoclonal antibody PAb101 specific for SV40 TAg was obtained from BD biosciences (Madrid, Spain). Monoclonal antibody specific for GAL4 DNA binding domain was obtained from Clontech (Saint Germain en Laye, Francia). Polyclonal antibody against p53 was obtained from Santa Cruz Biotechnology (Santa Cruz, California). The mouse monoclonal antibody (Mab) FK2 against conjugated ubiquitin [18] was from Biomol International L.P. (Exeter, United Kingdom). The following secondary antibodies were used as appropriate for different experiments: Alexa 594 and Alexa 488 conjugated antibodies against mouse and rabbit IgG, respectively (Sigma-Aldrich, Madrid, Spain).

### Transfections and IF microscopy

Transfections of cell monolayers were done with the Lipofectamine Plus reagent (Invitrogen, Barcelona, Spain), according to the manufacturer's instructions. Transfected cells were incubated at 37°C for 18 h, unless otherwise stated.

For indirect immunofluorescence microscopy, cell monolayers grown on coverslips were transfected, and, at the indicated times, the monolayers were washed twice with PBS and fixed for 10 min with 4% paraformaldehyde in PBS. Paraformaldehyde-fixed cells were washed twice with PBS, incubated for 4 min in permeabilizing buffer (0.5% Triton X-100 in PBS), and then blocked in PBS containing 2% bovine serum albumin for 1 h at room temperature. Then, the cells were incubated for 1 h at room temperature with primary antibodies diluted in blocking buffer. After three washes with PBS, the cells were incubated for 30 min with secondary antibodies and DAPI. Coverslips were then washed six times with PBS and mounted on glass slides. Images were obtained with an Olympus DP-71 digital camera mounted on an Olympus BX51 fluorescence microscope. Images were processed with Adobe Photoshop (Adobe Systems, California, USA).

### Immunoblotting

For Western-blot analysis, cell extracts were resolved by SDS-PAGE and proteins in unfixed gels were transferred to PVDF membranes (Immobilon-P Millipore, Madrid, Spain) for 1 h at 100 mA in a semidry blotting apparatus (Bio-Rad, California, USA). Protein bands were detected with specific antibodies using the Immobilon Western Chemiluminiscent HRP substrate (Millipore, Madrid, Spain).

### Plasmid constructions

The following plasmids have been described previously: i) pCINeo-muNS, which expresses full-length muNS [13]; ii) pCINeo-muNS(448–635) (termed muNS-Mi in the results), which expresses muNS residues 448-635 [14]; iii) pEGFP-C1M3-(448–635), which expresses GFP fused to muNS residues 448–635 (termed GFP-muNS-Mi in results) [14]; iv) pEGFP-C1-M3, which expresses GFP fused to the N terminus of muNS [13]; v) pVP16-muNS, which expresses a transcriptional activation domain (VP16) fused to the N terminus of µNS [14]; vi) GFP-muNS(477–542) (termed GFP-IC in results) [14]; viii) pCDNA3.1/Zeo-p53-muNS(477–542), which expresses p53-IC [25]; and vii) pCDNA3.1/Zeo-muNS(477–542) [25].

pCMV-wtTAg, which expresses full-length wild-type SV40 T Antigen, was a generous gift from Dr. J.B. Zalvide (University of Santiago de Compostela), and has been described previously [24].

*muNS-derived chimeras containing nuclear localization sequences were constructed as follows:*

#### i) TAg-NLS-muNS

To express the SV40 T antigen NLS (nuclear localization sequence) fused to the N terminus of muNS, the recombinant plasmid pGEMT-M3 [13] was subjected to PCR amplification with the following primers: the forward primer was 5′GCGGAATTCATCATGGGACCAAAGAAGAAGCGTAAAGTTATCATGGCGTCAACCAAGTGG-3′ (EcoRI site is single underlined and the start codon and SV40 T antigen NLS is double underlined) and the reverse primer was 5′GCGTCTAGA TCACAGATCATCCACCAATTCTTC-3′ (XbaI site is single underlined and the stop codon is double underlined). The PCR product was digested and cloned into the EcoRI and XbaI sites of pCDNA3.1/Zeo to generate pCDNA3.1/Zeo-TAg-NLS-muNS.

#### ii) TAg-NLS-GFP-muNS or TAg-NLS-GFP-muNS-Mi

To express the SV40 T antigen NLS fused to the N terminus of either GFP-muNS or GFP-muNS-Mi, the recombinant plasmids pEGFP-C1-M3 [13] and pEGFP-C1-M3(448–635) [14] were subjected to PCR amplification with the following primers: the forward primer was 5′GCGGGATCCATCATGGGACCAAAGAAGAAGCGTAAAGTTACCATGGTGAGCAAGGGCGAG-3′ (BamHI site is single underlined and the start codon and SV40 T antigen NLS is double underlined), and the reverse primer was 5′GCGTCTAGATCACAGATCATCCACCAATTCTTC-3′ (XbaI site is single underlined and the stop codon is double underlined). PCR products were digested and cloned into the BamHI and XbaI sites of pCDNA3.1/Zeo to generate either pCDNA3.1/Zeo-TAg-NLS-GFP-muNS or pCDNA3.1/Zeo-TAg-NLS-GFP-muNS-Mi.

#### iii) TAg-NLS-muNS-Mi

To express the SV40 T antigen NLS fused to the N terminus of muNS-Mi, the recombinant plasmid pGEMT-M3 [13] was subjected to PCR amplification with the following primers: the forward primer was 5′GCGGGATCCATCATGGGACCAAAGAAGAAGCGTAAAGTTCCAGCCGTACTGCTGTCTAAA-3′ (BamHI site is single underlined and the start codon and SV40 T antigen NLS is double underlined), and the reverse primer was 5′GCGTCTAGATCACAGATCATCCACCAATTCTTC-3′ (XbaI site is single underlined and the stop codon is double underlined). The PCR product was digested and cloned into the BamHI and XbaI sites of pCDNA3.1/Zeo to generate pCDNA3.1/Zeo-TAg-NLS-muNS-Mi.

#### iv) VP16-GFP-muNS or VP16-GFP-muNS-Mi

To generate the recombinant plasmids pVP16-GFP-muNS and pVP16-GFP-muNS-Mi, which express a transcriptional activation domain (VP16) fused to the N terminus of either GFP-muNS or GFP-muNS-Mi, the recombinant plasmids pEGFP-C1-M3 [13] and pEGFP-C1-M3(448–635) [14] were subjected to PCR amplification with the following primers: the forward primer was 5′-GCGGGATCCGTACCATGGTGAGCAAGGGCGAG-3′ (BamHI site is single underlined and the start codon is double underlined), and the reverse primer was 5′GCGTCTAGATCACAGATCATCCACCAATTCTTC-3′ (XbaI site is single underlined and the stop codon is double underlined). PCR products were cut with BamHI and XbaI and ligated to pVP16 (Clontech, Saint Germain en Laye, Francia) that had been cut with the same enzymes.

#### v) VP16-muNS-Mi

To generate the recombinant plasmid pVP16-muNS-Mi, which expresses a transcriptional activation domain (VP16) fused to the N terminus of muNS-Mi, the recombinant plasmid pGEMT-M3 [13] was subjected to PCR amplification with the following primers: the forward primer was 5′GCGGAATCCATCATGCCAGCCGTACTGCTGTCTAAA-3′ (EcoRI site is single underlined), and the reverse primer was 5′GCGTCTAGATCACAGATCATC CACCAATTCTTC-3′ (XbaI site is single underlined and the stop codon is double underlined). The PCR product was cut with EcoRI and XbaI and ligated to pVP16 (Clontech, Saint Germain en Laye, Francia) that had been cut with the same enzymes.

*3) GAL4-Intercoil:*

To generate the recombinant plasmid pM-muNS(477–542), which expresses the DNA-binding nuclear domain of GAL4 fused to the N terminus of muNS residues 477–542, the recombinant plasmid pGEMT-M3 [13] was subjected to PCR amplification with the following primers: the forward primer was: 5′GCGGAATTCATCATGGAAGATCACTTGTTGGCTTATC-3′ (EcoRI site is single underlined), and the reverse primer was: 5′GCGTCTAGATTACGCTTCCACACGGGGTTCCCAC-3′ (XbaI site is single underlined and the stop codon is double underlined). The PCR product was digested and cloned into the EcoRI and XbaI sites of pM (Clontech, Saint Germain en Laye, Francia) that had been cut with the same enzymes.

The correctness of the constructs was confirmed by sequencing and Western blot analysis of the expressed proteins.
